# Therapeutic strategies of thromboembolic events in patients with inflammatory bowel diseases: Two case reports

**DOI:** 10.1097/MD.0000000000014622

**Published:** 2019-03-01

**Authors:** Lei Zhu, Jiafei Cheng, Peiqing Gu, Yajun Liu, Junlou Liu, Jianhua Wang, Hong Shen

**Affiliations:** Department of Gastroenterology, Jiangsu Province Hospital of Chinese Medicine (Affiliated Hospital of Nanjing University of Chinese Medicine), Nanjing, China.

**Keywords:** Crohn disease, inflammatory bowel disease, thromboembolism, ulcerative colitis

## Abstract

**Rationale:**

Inflammatory bowel disease (IBD), including Crohn disease (CD) and ulcerative colitis (UC), is characterized by chronic inflammatory condition and immunological abnormalities, which probably develop into venous thromboembolic events (VTEs). VTE in IBD patients mostly occurs at deep venous thrombosis (DVT) and pulmonary embolism (PE). The complications are extremely important in clinical practice considering the high mortality rate. Hence, an early diagnosis of IBD and the control of complications play an important role in therapy of thromboembolic events (TEEs).

**Patient concerns:**

Case 1 was a 31-year-old man with chronic UC who presented with signs of thromboembolism. Case 2 was a 43-year-old woman with CD complicated by fistulas.

**Diagnoses:**

Computed tomography (CT) and digital subtraction angiography (DSA) of the patient (case 1) suggested a thrombus in cerebral vein. The patient (case 2) developed acute ischemia of her right arm; B ultrasonography revealed a thrombus in the distal of the right subclavian artery accompanied by stenosis.

**Interventions:**

To lower blood viscosity and overcome the risk of deep thrombosis, the patient (case 1) was treated with a combination of low-molecular-weight heparin and dextran as anticoagulation. For the patient (case 2), anticoagulation treatment with 75 mg qd clopidogrel (plavix) and 1.25 mg qd warfarin was performed.

**Outcomes:**

In both patients, no further TEE occurred during follow-up 1 year and one and a half years, respectively.

**Lessons:**

It is important to pay attention to IBD patients especially those with high coagulation state.

## 1 Introduction

Thromboembolic events (TEEs), both venous and arterial, are recognized complication of inflammatory bowel disease (IBD), represented by Crohn disease (CD) and ulcerative colitis (UC).^[1]^ IBD is characterized by chronic inflammation, and is accompanied by abnormal clotting and hypercoagulation.^[2]^ Previous report suggested that the risk of TEE in IBD is higher than that in the general population.^[3]^ TEE is a common severe complication of IBD arising in relatively younger patients, with the most frequent site being pulmonary vessels, the deep veins of the leg, and other sites such as hepatic, cerebral, and mesenteric vessels.^[4]^ The extent of colonic disease has a correlation with thromboembolic risk. Extensive UC and colonic involvement in CD were significantly associated with the development of thromboembolism.

Venous thromboembolism (VTE), and also arterial thromboembolism, is accompanied by the incidence and mortality of CD and UC. VTE, which comprises pulmonary embolism (PE) and deep vein thrombosis (DVT) of lower limbs, represents a significant worldwide health problem that can lead to death. A solid evidence has confirmed that the risk of VTE was 8 times higher in IBD patients than in healthy individuals.^[5]^ Several acquired thrombotic risk factors may be present in IBD including prolonged immobilization, fluid depletion, and smoking. More than half of the cases of VTE in IBD possibly be associated with prothrombin gene mutation and factor V Leiden, which may reveal that genetic factors play a role in TEE.^[6]^ However, the detailed mechanisms remain controversial. Herein, we report 2 cases of thromboembolism associated with IBD.

## 2 Case reports

### 2.1 Case 1

**Figure 1:**
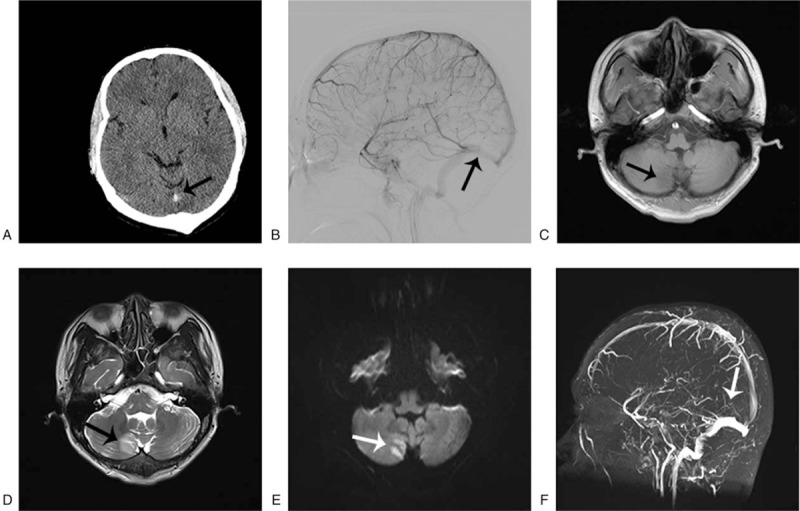
(A) Noncontrast cerebral CT scan showing spontaneous hyperdensity of the straight sinus (black arrow). (B) Straight sinus (black arrow) is not seen on cerebral DSA, confirming thrombosis. (C–E) T1-weighted MRI, T2-weighted MRI and DWI showing an acute infarction at the right cerebellar hemisphere (black arrow). (F) Noncontrast magnetic resonance venography showing the defect in the vein of the straight sinus (white arrow). CT = computed tomography, DSA = digital subtraction angiography, MRI = magnetic resonance imaging.

**Figure 2:**
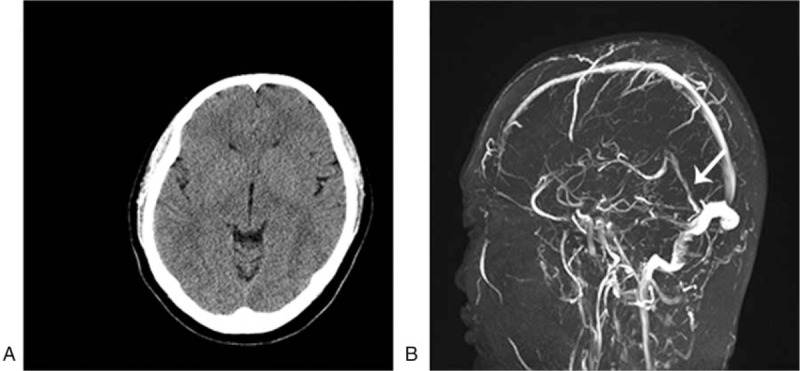
(A) Noncontrast cerebral CT scan showing the original dense clot sign of straight sinus has disappeared. (B) Straight sinus is seen on cerebral MRV (white arrow). CT = computed tomography, MRV = magnetic resonance venography.

A 31-year-old man presented with a 7-year history of UC, which had chronic recurrence, was moderate, in active stage, and in the full colon. He used azathioprine and hormones to relieve the discomfort for a long term. He denied other previous diseases including hypertension, diabetes, coronary heart disease, smoking, and drinking, and his family history did not reveal any relevant pathological elements. The patient was admitted to our hospital because of bloody diarrhea. On admission, the clinical examination showed the following pathological elements: hemoglobin (Hb) level (106 g/L), platelet count (PLT) (465 × 10^9^/L), erythrocyte sedimentation rate (ESR) (30 mm/H), prothrombin time (PT) (32.20 seconds), international normalized ratio 2.96, and D-dimer (1.12 mg/L). During hospitalization, the patient started complaining of a headache accompanied with nausea and vomiting, whereas blood pressure (BP) was within the normal range. A computed tomography (CT) of the head suggested focal high density in straight sinus, digital subtraction angiography (DSA) under local anesthesia showed no signs in straight sinus and bilateral transverse sinus of brain, which were highly suggestive of a thrombus (Fig. 1A and B). Brain magnetic resonance (MR) and magnetic resonance venography (MRV) imaging revealed abnormal signs in the right cerebella hemisphere, low T1-weighted imaging and apparent diffusion coefficient signal intensity, high T2-weighted imaging, fluid-attenuated inversion recovery, and diffusion-weighted imaging (DWI) MR signal intensity (Fig. 1C–F), demonstrating thrombosis of cerebral venous sinus. To reduce blood viscosity and overcome the risk of deep thrombosis, the patient was treated with a combination of low-molecular-weight heparin and dextran for anticoagulation. The patient was subsequently transitioned to edaravone, tropisetron, and valproate to improve neurological function, vomiting, and prevent epilepsy, respectively. After treatment, the clinical symptoms (headache and vomiting) were improved notably. Meanwhile, the blood coagulation of the patient was strictly examined during therapy. Anticoagulant therapy with warfarin (5 mg/d, oral) was continued after 1 week. One-year follow-up MRV and CT showed no signs of thrombosis recurrence (Fig. 2A and B).

### 2.2 Case 2

**Figure 3:**
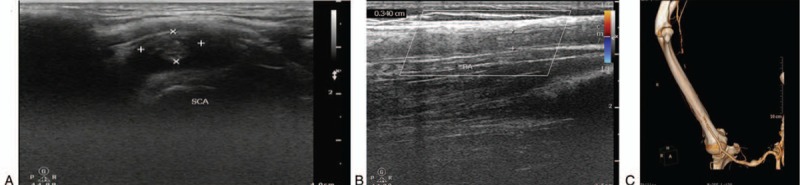
(A) Thrombosis of the right subclavian artery distant with stenosis (diameter stenosis rate was 77.1%). (B) Poor filling of local blood in the right brachial artery, thrombosis was not excluded. (C) CTA of the right upper limb: aneurysm occurred in the right subclavian, thrombosis of the right brachial artery medium-to-distal. CTA = computed tomography angiography.

**Figure 4:**
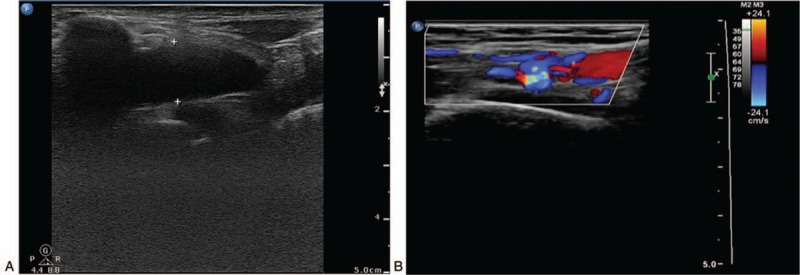
(A) Thrombosis review of subclavian artery showed remarkably reduced lesion (6 months after surgery). (B) Thrombus organization after brachial artery, variegated blood flow filling was seen (one and a half years after surgery).

A 43-year-old woman with medical history of CD had been treated with mesalazine and infliximab, and had undergone surgical history of anal fistula earlier. Her only regular medications were thalidomide, prednisone, and azathioprine tablets successively within 6 months before admission. Two months before this present admission, the patient developed intermittent dystonic posture of the right arm and significantly decreased skin temperature without obvious incentive. On examination, the patient's BP of the left arm was 101/73 mm Hg, whereas BP of the right one could not be measured. The skin temperature of the right upper limb was significantly lower than that of the left one, and pulse of the right brachial artery and radial artery were weakened. Routine tests on admission revealed Hb 87 g/L, PLT 425 × 10^9^/L, ESR 20 mm/h, and C-reactive protein (CRP) 14.1 mg/L. Laboratory tests were performed to diagnose possible CD, which was ileal fatty acid binding protein and remission, and the cause of right upper limb weakness remained to be determined. After admission, B ultrasonography of the right upper arm arteriovenous depicted poor filling of local blood in the right brachial artery. Based on this, thrombosis was not ruled out. Furthermore, B ultrasonography of carotid artery displayed thrombosis of the right subclavian artery distant with stenosis (diameter stenosis rate was 77.1%) (Fig. 3A and B). Taken together, thrombosis of the right subclavian artery and brachial artery was confirmed. After anticoagulation treatment with 75 mg qd clopidogrel (plavix) and 1.25 mg qd warfarin, local tumor-like dilatation of the right subclavian artery accompanied with thrombosis was observed, and the lateral branch circulation of right brachial artery thrombosis was established by B ultrasonography. The patient was prepared to surgical thrombectomy after communication. The patient received thrombectomy and vascular nerve exploration to block the distal and proximal of brachial artery. A 0.5-cm long transverse cut was taken on the anterior wall of the transverse artery, then local thrombosis was formed and bleeding between the proximal and distal was not found (Fig. 3C). Plenty of hoary old thrombus and dark red mixed thrombus was fetched by blocker catheter; the proximal spray was good after surgery and blood flow from distal was better. Postoperative pathology was thrombosis accompanied by mechanization (Fig. 4A and B), and there was no repeating of symptoms. Warfarin and azathioprine treatment was continued to prevent coagulation and maintain CD remission.

### 2.3 Patient anonymity and informed consent

The patients involved gave their permission for the publication of the case reports.

## 3 Discussion

Inflammatory bowel disease is a chronic inflammatory disorder of the intestine with morbidity of 6% to 47%. TEE with an incidence rate of 1% to 10% is critical extraintestinal manifestations among IBD. Previous report has shown that the risk of TEE in IBD patients is increased regardless of diagnosis (CD or UC) compared with normal individuals, no matter whether IBD pathology is in active state or quiescent.^[7]^ TEE in IBD patients occurs more often in the deep veins, and DVT and PE are the most common ones with the rate of 90.4%. IBD patients have about 3-fold higher risk of VTE when compared with the non-IBD population, and may involve in the thrombosis of the retinal, coronary, splanchnic, cerebral, carotid, and the aorta.^[8]^ Hospitalization is an independent risk factor for VTE in IBD patients. A study found that the overall incidence of VTE in IBD patients was 24 per 1000 person-years while hospitalized.^[9]^

Pathogenesis of TEE associated with IBD was complicated and not completely known.^[8]^ There had been several genetic polymorphism studies on the risk of thrombosis with IBD, while related genomic risk markers have not been reported.^[9]^ The most common genetic mutation that promoted thrombosis was G1691A mutant of the encoding of clotting factors V, and other mutations were unusual.^[10,11]^ Existing reports revealed that UC patients exhibited abnormal platelet, coagulation, and fibrinolytic system, and increased PLT. Among IBD patients, 30% to 50% occurred platelet aggregation spontaneously or hypersensitivity to low concentration of stimulus.^[12]^ Investigators depicted that the levels of some coagulation enzymes like fibrinogen, factors V, VII, VIII, X, XI, and XII, prothrombin fragment 1 + 2, and the thrombin-antithrombin complex (TAT) were changed in IBD patients.^[13]^ Normally, endothelial cells provide an antiadhesive and selectively permeable exchange barrier. In IBD, endothelial dysfunction plays an important role in the production of a hypercoagulable state due to the effects of proinflammatory cytokines. It was demonstrated that the impaired endothelial cell in IBD patients was associated with increased expression of endothelial protein C receptor, thrombomodulin and disease activity, and inflammatory markers.^[2,14]^ Inflammation and the coagulation system interacted with each other, and an in vitro study had confirmed that inflammation could disturb the balance of coagulation, activate the system of coagulation, then promote thrombosis. Reversely, activation of the coagulation system caused continuous inflammation. The mechanism of thrombosis with inflammation was complex, for example, tumor necrosis factor-alpha (TNF-α) and CRP could induce white blood cells to express tissue factors and activate endogenous coagulation, TNF-α and interleukin 1 beta inhibited the anticoagulation system of protein C and antithrombin III, CRP suppressed fibrinolytic activity, and thus promoted thrombosis.^[15,16]^ The antiphospholipid antibodies including lupus anticoagulant, anticardiolipin (aCL) antibodies, and antiphosphatidic acid antibody had positive effect on thrombosis. In a recent study, aCL antibody titers were remarkably increased in patients with CD compared with controls; however, the exact relationship between aCL antibody and thrombosis remained unclear.^[17]^

Glucocorticoid was a risk factor for thrombosis with the mechanisms of enhancing coagulation and suppressing fibrinolysis, which may be contributed to the increase of fibrinogen and inhibition of tissue plasminogen activator.^[18]^ Hormones reduced thrombus through inhibiting inflammation and partial compensation for thrombotic effects; the relative risk of thrombosis was 1.87.^[19]^

Clinical study suggested that treatment of TEE in IBD patients had no difference with normal individuals, which covered risk factors removing, anticoagulant thrombolysis activating, and surgical treatment.^[7]^ In 2014, Canadian Association of Gastroenterology Statements summarized the 17 recommendation statements for the treatment of venous thrombosis in IBD patients, and 3 were about morbidity, 9 for prevention, and the other 5 for treatment. The consensus noted moderately severe and active IBD was the risk of venous thromboembolism, and IBD patients should take actively anticoagulation treatment.^[20]^ This consensus will have good directive significance for the diagnosis and treatment of TEE patients with IBD. At present, the incidence of IBD is increasing yearly; cases of thrombosis have been reported recently, but the incidence and effective treatment of thrombosis were rarely reported.

## 4 Conclusions

In conclusion, IBD patients, especially those with high coagulation state and dangerous factors such as pregnancy, birth control pills, and medication history, should be paid more attention. Early prevention, diagnosis, and treatment are necessary for avoiding serious clinical incident.

## Author contributions

**Validation:** Hong Shen.

**Writing – original draft:** Lei Zhu, Jiafei Cheng.

**Writing – review & editing:** Peiqing Gu, Yajun Liu, Junlou Liu, Jianhua Wang.

Hong Shen orcid: 0000-0001-9446-6896.
